# Multi-drug resistance, inappropriate initial antibiotic therapy and mortality in Gram-negative severe sepsis and septic shock: a retrospective cohort study

**DOI:** 10.1186/s13054-014-0596-8

**Published:** 2014-11-21

**Authors:** Marya D Zilberberg, Andrew F Shorr, Scott T Micek, Cristina Vazquez-Guillamet, Marin H Kollef

**Affiliations:** EviMed Research Group, LLC, PO Box 303, Goshen, MA 01032 USA; University of Massachusetts, PO Box 303, Amherst, MA USA; Washington Hospital Center, 110 Irving St NW, Washington, DC 20010 USA; St. Louis College of Pharmacy, 4588 Parkview Place, St. Louis, MO 63110 USA; University of New Mexico School of Medicine, Department of Medicine, MSC 10 5550, 1 University of New Mexico, Albuquerque, NM 87131 USA; Division of Pulmonary and Critical Care Medicine, Washington University School of Medicine, 660 South Euclid Avenue, Campus Box 8052, St. Louis, MO 63110 USA

## Abstract

**Introduction:**

The impact of *in vitro* resistance on initially appropriate antibiotic therapy (IAAT) remains unclear. We elucidated the relationship between non-IAAT and mortality, and between IAAT and multi-drug resistance (MDR) in sepsis due to Gram-negative bacteremia (GNS).

**Methods:**

We conducted a single-center retrospective cohort study of adult intensive care unit patients with bacteremia and severe sepsis/septic shock caused by a gram-negative (GN) organism. We identified the following MDR pathogens: MDR *P. aeruginosa*, extended spectrum beta-lactamase and carbapenemase-producing organisms. IAAT was defined as exposure within 24 hours of infection onset to antibiotics active against identified pathogens based on *in vitro* susceptibility testing. We derived logistic regression models to examine a) predictors of hospital mortality and b) impact of MDR on non-IAAT. Proportions are presented for categorical variables, and median values with interquartile ranges (IQR) for continuous.

**Results:**

Out of 1,064 patients with GNS, 351 (29.2%) did not survive hospitalization. Non-survivors were older (66.5 (55, 73.5) versus 63 (53, 72) years, *P* = 0.036), sicker (Acute Physiology and Chronic Health Evaluation II (19 (15, 25) versus 16 (12, 19), *P* <0.001), and more likely to be on pressors (odds ratio (OR) 2.79, 95% confidence interval (CI) 2.12 to 3.68), mechanically ventilated (OR 3.06, 95% CI 2.29 to 4.10) have MDR (10.0% versus 4.0%, *P* <0.001) and receive non-IAAT (43.4% versus 14.6%, *P* <0.001). In a logistic regression model, non-IAAT was an independent predictor of hospital mortality (adjusted OR 3.87, 95% CI 2.77 to 5.41). In a separate model, MDR was strongly associated with the receipt of non-IAAT (adjusted OR 13.05, 95% CI 7.00 to 24.31).

**Conclusions:**

MDR, an important determinant of non-IAAT, is associated with a three-fold increase in the risk of hospital mortality. Given the paucity of therapies to cover GN MDRs, prevention and development of new agents are critical.

## Introduction

Antimicrobial resistance is a growing challenge in the care of critically ill patients, among whom the burden of infection remains high. Escalating rates of antibiotic resistance add substantially to the morbidity, mortality, and cost related to infection in the ICU [1]. Traditionally, most efforts to understand issues of resistance and ICU outcomes have addressed Gram-positive organisms, such as methicillin-resistant *Staphylococcus aureus* [2,3]. However, in the United States, alarming trends in resistance are now also reported for a number of Gram-negative pathogens. For example, extended-spectrum beta-lactamase (ESBL) organisms are now endemic in many ICUs, and 15 to 20% of all *Pseudomonas aeruginosa* isolates from serious infections are categorized as multidrug resistant (MDR) because of reduced *in vitro* susceptibility to three or more classes of antibiotics [4-6]. Of even more concern are pathogens for which clinicians have few antibiotic options, namely *Acinetobacter baumanii* and carbepenemase-producing *Enterobacteriaceae* (CPE) [4-6]. In the case of these Gram-negative organisms, studies also point to an association between resistance and both clinical and economic outcomes [1].

The mechanism for poor outcomes with resistant Gram-negative organisms is not completely clear. In general, these bacteria are not believed to be inherently more virulent than similar susceptible species. Resistance and its rapid evolution, however, make efforts to insure initially appropriate antibiotic therapy (IAAT) more difficult, and IAAT is a key determinant of outcome in severe infection [7-10]. IAAT has consistently been shown to reduce mortality rates in severe sepsis and septic shock, and the Surviving Sepsis Campaign Guidelines strongly support initiatives to guarantee that patients receive timely antibiotic treatment [11-16]. However, it remains unclear what proportion of IAAT is driven by *in vitro* resistance. Appreciating this relationship may facilitate efforts to improve outcomes by helping clinicians determine how to apply newer diagnostic modalities and therapeutic options.

We sought to confirm the importance of IAAT in severe sepsis and septic shock due to Gram-negative bacteria and to estimate the impact of initially inappropriate antibiotic therapy (non-IAAT) on mortality in these syndromes. More importantly, we aimed to identify variables associated with IAAT and to elucidate the relationship between IAAT and *in vitro* antimicrobial resistance. To accomplish this we conducted a large retrospective analysis of subjects with severe sepsis or septic shock and Gram-negative bacteremia.

## Materials and methods

### Study design and ethical standards

We conducted a single-center retrospective cohort study from January 2008 to December 2012. Barnes-Jewish Hospital is a 1,200-bed urban academic medical center located in St. Louis, MO, USA. The study was approved by the Washington University School of Medicine Human Studies Committee and informed consent was waived since the data collection was retrospective without any patient-identifying information. The study was performed in accordance with the ethical standards of the 1964 Declaration of Helsinki and its later amendments.

### Study cohort

All consecutive adult ICU patients between January 2008 and December 2012 were included if: they had a positive blood culture for a Gram-negative organism; and there was an International Classification of Diseases, Version 9, Clinical Modification (ICD-9-CM) code corresponding to an acute organ dysfunction [17]. Only the first episode of sepsis was included.

### Definitions

To be included in the analysis, patients had to meet criteria for severe sepsis based on discharge ICD-9-CM codes for acute organ dysfunction [17]. Patients were classified as having septic shock if vasopressors (norepinephrine, dopamine, epinephrine, phenylephrine, or vasopressin) were initiated within 24 hours of the blood culture collection date and time. Antimicrobial treatment was deemed IAAT if the initially prescribed antibiotic regimen was active against the identified pathogen based on *in vitro* susceptibility testing and was administered within 24 hours following blood culture collection. Combination therapy was not required to be considered IAAT. We also required that antibiotics had to be prescribed for at least 24 hours. All other regimens were classified as non-IAAT. Prior antibiotic exposure was any exposure to an antibiotic within the preceding 90 days. Combination antimicrobial treatment was not required for IAAT designation. This is supported by multiple studies indicating that while dual therapy is more likely than single therapy to result in appropriate coverage, it is not necessarily associated with better outcomes provided the organism is adequately covered by a single drug [18]. We utilized the same time frame (90 days prior to the onset of the current episode of bacteremia) to define prior hospitalization. In contrast, prior bacteremia was defined by a bacteremia that had occurred within 30 days of the current episode. Multidrug-resistant *P. aeruginosa* (MDR-PA) was defined as *P. aeruginosa* resistant to at least three of the following classes of antimicrobials: aminoglycosides, anti-pseudomonal penicillins, anti-pseudomonal cephalosporins, carbapenems, and fluoroquinolones. A case was classified as MDR if the blood culture was positive for a MDR-PA, an ESBL organism, or a CPE. Both ESBL and CPE status were established based on molecular laboratory testing.

### Antimicrobial treatment algorithms

From January 2002 through to the present, Barnes-Jewish Hospital utilized an antibiotic control program to help guide antimicrobial therapy. During this time cefepime, gentamicin, vancomycin, or fluconazole use was unrestricted. However, initiation of ciprofloxacin, imipenem, meropenem, piperacillin/tazobactam, linezolid, daptomycin, or micafungin was restricted and required preauthorization from a clinical pharmacist or infectious diseases physician. Each ICU had a clinical pharmacist who reviewed antibiotic orders to ensure that dosing and the interval of administration were adequate for patients based on body size, renal function, and resuscitation status. After daytime hours the on-call clinical pharmacist reviewed and approved the antibiotic orders. The initial antibiotic dosages employed for treatment were as follows: cefepime, 1 to 2 g every 8 hours; piperacillin-tazobactam, 4.5 g every 6 hours; imipenem, 0.5 g every 6 hours; meropenem, 1 g every 8 hours; ciprofloxacin, 400 mg every 8 hours; gentamicin, 5 mg/kg once daily; vancomycin, 15 mg/kg every 12 hours; linezolid, 600 mg every 12 hours; daptomycin, 6 mg/kg every 24 hours; fluconazole, 800 mg on the first day followed by 400 mg daily; and micafungin, 100 mg daily.

Starting in June 2005, with regular updates, a sepsis order set was implemented in the emergency department, general wards, and the ICUs with the intent of standardizing empiric antibiotic selection for patients with sepsis based on the infection type (i.e. community-acquired pneumonia, healthcare-associated pneumonia, intra-abdominal infection, and so forth) and the hospital’s antibiogram. However, antimicrobial selection, dosing, and de-escalation of therapy were still optimized by clinical pharmacists in these clinical areas.

### Antimicrobial susceptibility testing

The microbiology laboratory performed antimicrobial susceptibility of the Gram-negative blood isolates using the disk diffusion method according to guidelines and breakpoints established by the Clinical Laboratory and Standards Institute and published during the inclusive years of the study [19,20].

### Data elements

Patient-specific baseline characteristics and process of care variables were collected from the automated hospital medical record, microbiology database, and pharmacy database of Barnes-Jewish Hospital. Electronic inpatient and outpatient medical records available for all patients in the BJC Healthcare system were reviewed to determine prior antibiotic exposure. The baseline characteristics collected included: age, gender, race, past history of congestive heart failure, chronic obstructive pulmonary disease, diabetes mellitus, chronic liver disease, underlying malignancy, and end-stage renal disease requiring dialysis. The comorbidities were identified based on their corresponding ICD-9-CM codes. The Acute Physiology and Chronic Health Evaluation II and Charlson comorbidity scores were calculated based on clinical data present during the 24 hours after the positive blood cultures were obtained [21]. This was done to accommodate patients with community-acquired and healthcare-associated community-onset infections who only had clinical data available after blood cultures were drawn. Healthcare-associated infections were defined by the presence of at least one of the following risk factors: recent hospitalization (within 90 days of the current one); immune suppression; nursing home residence; hemodialysis; and prior antibiotics (within 90 days of the current hospitalization). The primary outcome variable was hospital mortality. Because we were interested in understanding the contribution of MDR pathogens to the risk of receiving non-IAAT, we examined this variable as a secondary endpoint in a logistic regression.

### Statistical analyses

Continuous variables were reported as means with standard deviations and as medians with 25th and 75th percentiles. Differences between mean values were tested via the Student’s *t* test, while those between medians were examined using the Mann–Whitney *U* test. Categorical data were summarized as proportions, and the chi-square test or Fisher’s exact test for small samples was used to examine differences between groups. We developed several multiple logistic regression models to identify clinical risk factors that were associated with hospital mortality. In the mortality models, all risk factors that were significant at ≤0.20 in the univariate analyses, as well as all biologically plausible factors even if they did not reach this level of significance, were included in the corresponding multivariable analyses. All variables entered into the models were examined to assess for colinearity, and interaction terms were tested. The most parsimonious models were derived using the backward manual elimination method, and the best-fitting model was chosen based on the area under the receiver operating characteristics curve (*c* statistic). The model’s calibration was assessed with the Hosmer–Lemeshow goodness-of-fit test. Similarly, the most parsimonious model for the predictors of inappropriate empiric antibiotic was computed and its fit was tested with the *c* statistic and the Hosmer–Lemeshow goodness of fit. All tests were two tailed, and *P* <0.05 was deemed *a priori* to represent statistical significance.

All computations were performed in Stata/SE, version 9 (StataCorp, College Station, TX, USA).

## Results

In total, 1,076 patients with severe sepsis or septic shock due to a Gram-negative pathogen met the inclusion criteria. The distribution of the pathogens is presented in Table 1. Among these 1,076 culture-positive cases, there were 63 (5.9%) cultures that met the MDR criteria (Table 1). The most common MDR organism was MDR-PA, accounting for 15.0% of all *P. aeruginosa* isolates.

**Table 1:** **Microbiology of Gram-negative severe sepsis and septic shock**

	**All organisms**		**MDR-PA**		**ESBL**		**CP**		**Total MDR**	
	***N***	**%**	***N***	**%**	***N***	**%**	***N***	**%**	***N***	**%**
*Pseudomonas aeruginosa* ^a^	173	16.08	26	15.03	1	0.58	1	0.58		
*Acinetobacter* spp.^b^	73	6.78			1	1.37	1	1.37		
*Bacteroides* spp.	83	7.71								
*Stenotrophomonas maltophilia*	22	2.04								
*Enterobacteriaceae*										
*Klebsiella pneumoniae* ^c^	217	20.17			13	5.99	8	3.69		
*Escherichia coli*	284	26.39			14	4.93				
*Klebsiella oxytoca*	35	3.25			3	8.57				
*Proteus mirabilis*	55	5.11								
*Serratia marcescens*	46	4.28								
*Citrobacter freundii*	25	2.32								
*Enterobacter aerogenes*	35	3.25								
*Enterobacter cloacae*	90	8.36			1	1.11				
Other^d^	6	0.56								
Polymicrobial	191	17.75								
Total	1,076	100.00	26		33^e^		10		63^f^	5.86

CP, carbapenemase-producing; ESBL, extended-spectrum beta lactamase; MDR, multidrug resistant; MDR-PA, multidrug resistant *P. aeruginosa*. ^a^Same MDR-PA specimen that was positive for both ESBL and CP. ^b^Same *Acinetobacter baumanii* specimen that was positive for both ESBL and CP. ^c^Two patients each had one CP *K. pneumoniae* + one ESBL *K. pneumoniae*. ^d^ *Aeromonas sobria* (*n* = 2), *Haemophilis influenza* (*n* = 2), *Pseudomonas putida* (*n* = 1), *Achromobacter* sp. (*n* = 1). ^e^These 33 specimens came from 32 patients (one patient had 2 ESBL organisms: *E. coli* and *K. pneumoniae*). ^f^The six-sample discrepancy is explained by the above overlaps, and one patient has ESBL *E. coli* and CP *K. pneumoniae*.

Among the 1,064 patients whose hospital disposition was known, 311 (29.2%) died in the hospital. Their baseline characteristics are presented in Table 2. Patients who died were older, less likely to be admitted from home, and had a higher comorbidity burden than those who survived their hospitalization, as signified by the Charlson comorbidity score. A higher proportion of those patients who died prior to discharge (95.7%) had a risk factor for a healthcare-associated infection than those who were discharged alive (91.4%, *P* = 0.014).

**Table 2:** **Baseline and infection characteristics and outcomes**

	**Died (** ***n*** **= 311)**		**Survived (** ***n*** **= 753)**		***P*** **value**
	***N***	**%**	***N***	**%**	
**Baseline characteristics**					
Age (years)					
Mean ± standard deviation	65.0 ± 13.0			62.3 ± 14.8	
Median (25th, 75th percentiles)	66.5 (55, 73.5)			63 (53, 72)	0.036
Race					
Caucasian	198	63.67	504	66.93	0.101
African-American	96	30.87	193	25.63	
Hispanic	0	0.00	1	0.13	
Other	1	0.32	8	1.06	
Unknown	10	3.22	41	5.44	
Asian	6	1.93	6	0.80	
Sex, female	140	45.60	356	47.34	0.518
Admission source					
Home	178	57.23	530	70.48	0.001
Nursing home/LTAC	30	9.65	62	8.24	
Transfer from other hospital	88	28.30	143	19.02	
Unknown	14	4.50	14	1.86	
Other	1	0.13	3	0.40	
Comorbidities					
CHF	78	25.08	136	18.06	0.009
COPD	92	29.58	171	22.71	0.018
CLD	65	20.90	105	13.94	0.005
DM	79	25.40	195	25.90	0.867
CKD	68	21.86	126	16.73	0.049
Malignancy	128	41.16	340	45.15	0.232
HIV	6	1.93	6	0.80	0.112
Charlson comorbidity score					
Mean ± standard deviation	5.4 ± 3.6		4.9 ± 3.3		
Median (25th, 75th percentiles)	5 (3, 8)		4 (2, 7)		0.022
HCA risk factors	292	95.74	676	91.35	0.014
Hemodialysis	41	13.62	52	6.92	0.001
Immune suppression	134	44.08	290	39.30	0.153
Prior hospitalization	204	69.86	445	62.06	0.019
Nursing home residence	30	9.65	62	8.23	0.456
Prior antibiotics	194	62.38	405	53.78	0.010
Hospital-acquired BSI^a^	153	49.20	350	46.48	0.420
Bacteremia that was not HCA (that is, community acquired)	19	6.11	77	10.23	0.033
Prior bacteremia within 30 days	37	11.90	97	12.88	0.660
**Sepsis characteristics and outcomes**					
LOS prior to sepsis onset, days					
Mean ± standard deviation	9.8 ± 18.4		7.3 ± 12.1		
Median (25th, 75th percentiles)	2 (0, 13)		1 (0, 11)		0.227
Surgery					
None	227	73.94	510	68.36	0.011
Abdominal	38	12.38	150	20.11	
Extra-abdominal	42	13.68	86	11.53	
Central line	199	67.46	462	63.55	0.236
Total parenteral nutrition	19	6.33	56	7.53	0.499
APACHE II score					
Mean ± standard deviation	19.9 ± 7.4		15.8 ± 5.4		
Median (25th, 75th percentiles)	19 (15, 25)		16 (12, 19)		<0.001
Peak WBC					
Mean ± standard deviation	21.6 ± 18.7		22.6 ± 17.7		
Median (25th, 75th percentiles)	16.4 (7.2, 32)		18.0 (8.2, 37)		0.275
Infection source^b^					
Urine	60	19.29	201	26.69	0.011
Abdomen	49	15.76	106	14.08	0.48
Lung	88	28.30	129	17.13	<0.001
Line	23	7.40	86	11.42	0.049
CNS	4	1.29	3	0.40	0.204
Skin	20	6.43	42	5.58	0.589
Unknown	90	28.94	241	32.01	0.326
Polymicrobal	60	19.29	129	17.13	0.402
Total hospital LOS (days)					
Mean ± standard deviation	22.9 ± 28.3		23.3 ± 23.7		
Median (25th, 75th percentiles)	15 (6, 28)		17 (8, 30)		0.013
Hospital LOS following sepsis onset, days					
Mean ± standard deviation	13.1 ± 19.8		16.0 ± 18.0		
Median (25, 75)	8 (3, 17)		10 (6, 20)		<0.001

APACHE, Acute Physiology and Chronic Health Evaluation; BSI, bloodstream infection; CHF, congestive heart failure; CKD, chronic kidney disease; CNS, central nervous system; COPD, chronic obstructive pulmonary disease; CLD, chronic liver disease; DM, diabetes mellitus; HCA, healthcare-associated; LOS, length of stay; LTAC, long-term acute care; WBC, white blood cells. ^a^Hospital-acquired BSI defined as BSI that developed after day 2 of hospitalization. ^b^Multiple sources possible.

**Figure 1:**
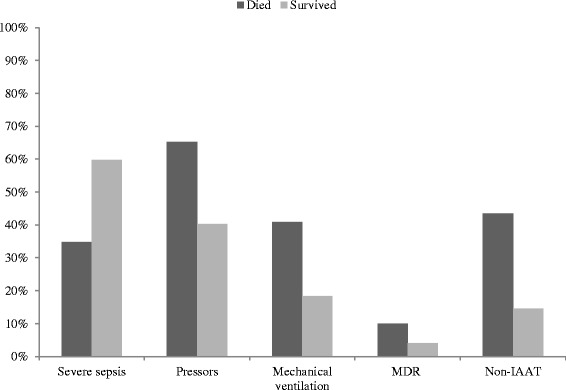
**Sepsis severity, resistance and initial treatment.** IAAT, initially appropriate antibiotic therapy; MDR, multidrug resistant. *P* <0.001 for each comparison.

In the run-up to and at the time of sepsis onset, patients who did not survive had a slightly longer presepsis hospital length of stay, although this difference did not meet the predetermined level of statistical significance (Table 2). Several healthcare-associated factors (hemodialysis, prior hospitalization, and antibiotics) were more prevalent among nonsurvivors. However, the vast majority of the cohort (over 90%) had at least one healthcare-associated risk factor (Table 2). Additionally, survivors had a higher frequency of having had surgery during the index hospitalization than those who died. All markers of severity of acute illness were higher in patients who died compared with those who survived; the Acute Physiology and Chronic Health Evaluation II score was higher, and septic shock and the need for mechanical ventilation were significantly more prevalent among nonsurvivors than among survivors (Table 2, Figure 1). Urine and an infected line were less likely sources of infection and the lung was more likely as a source of infection among nonsurvivors compared with survivors. There were also striking differences between the two groups in terms of the likelihood of a MDR pathogen as the sepsis culprit (10.0% among nonsurvivors vs. 4.0% among survivors, *P* <0.001) (Figure 1). Additionally, nonsurvivors were approximately three times more likely to receive non-IAAT than those patients who survived their hospitalization (43.4% vs. 14.6%, *P* <0.001) (Figure 1). Among the 245 patients who received non-IAAT, resistance to instituted empiric therapy was far more prevalent as a reason (75.5%) than delay in treatment (24.5%). When stratified by hospital death, the relationship generally held, although delay in treatment was slightly more likely among those who died (28.9%) than those who survived their hospitalization (19.1%, *P* = 0.076). Similarly, delay in therapy accounted for a minority of non-IAAT among patients with a MDR pathogen (25.5%), with a nearly identical frequency of delay observed among those without a MDR pathogen (23.8%, *P* = 0.798).

Multiple logistic regression models were constructed and tested for fit, with the factors presented in Table 3 having the best discrimination. In this model, as in others that included this model, receiving non-IAAT was the strongest predictor of hospital death with an adjusted odds ratio of 3.87 (95% confidence interval = 2.77 to 5.41, *P* <0.001, *c* statistic = 0.777).

**Table 3:** **Predictors of hospital mortality** ^**a**^

	**Odds ratio**	**95% confidence interval**	***P*** **value**
Non-IAAT	3.872	2.770 to 5.413	<0.001
Chronic liver disease	1.942	1.319 to 2.860	0.001
Septic shock	1.846	1.335 to 2.553	<0.001
Pneumonia	1.766	1.237 to 2.522	0.002
Mechanical ventilation	1.669	1.172 to 2.376	0.005
APACHE II score (per 1 point)	1.076	1.047 to 1.105	<0.001
Surgery	0.701	0.560 to 0.879	0.002
Admitted from home	0.677	0.489 to 0.936	0.018
Urosepsis	0.675	0.469 to 0.972	0.034

^a^Independent variables included but not retained in the model at alpha ≤0.05: age, race, admission sources other than home (nursing home or transfer from another facility), comorbidities of congestive heart failure, chronic obstructive pulmonary disease, chronic kidney disease and human immune deficiency virus infection, Charlson comorbidity score, healthcare-associated infection risk factors (hemodialysis, immune suppression, prior hospitalization, prior antibiotics), mechanical ventilation, and infection source other than urine (lung, abdomen, line, central nervous system, skin). Variables pressors and severe sepsis were excluded because of collinearity with septic shock. APACHE, Acute Physiology and Chronic Health Evaluation; IAAT, initially appropriate antibiotic therapy. Area under the receiver operating characteristics curve = 0.777; Hosmer–Lemeshow *P* = 0.823.

When focusing on the choice of empiric treatment among patients with a MDR pathogen versus those without, the unadjusted odds ratio of receiving non-IAAT was 11.79 (95% confidence interval = 6.55 to 21.23, *P* <0.001). In a logistic regression model to examine the factors that contribute to this inappropriate choice of therapy, a MDR pathogen as the etiology of sepsis was the strongest predictor of inappropriate treatment with an adjusted odds ratio of 13.05 (95% confidence interval = 7.00 to 24.31, *P* < 0.001, *c* statistic = 0.738) (Table 4). This parameter had by far the highest odds of any variable retained in the model of predictors of non-IAAT. (Tables 5, 6 and 7 present the details of characteristics based on appropriateness of treatment, as well as an alternative model for the predictors of non-IAAT. See Table 7 footnote for a brief discussion of that model.)

**Table 4:** **Predictors of receiving initially inappropriate antibiotic therapy** ^**a**^

	**Odds ratio**	**95% confidence interval**	***P*** **value**
Multidrug resistant	13.05	7.00-24.31	<0.001
HIV	3.64	1.02-12.95	0.046
Transferred from another hospital	2.86	2.00-4.08	<0.001
Nursing home resident	2.28	1.35-3.84	0.002
Prior antibiotics	2.06	1.47-2.87	<0.001
Polymicrobial	1.90	1.30-2.77	0.001
Congestive heart failure	1.61	1.11-2.35	0.013
APACHE II score (per 1 point)	1.05	1.02-1.07	<0.001

^a^Independent variables included but not retained in the model at alpha ≤0.05: age, admission source other than transfer from another hospital (home or nursing home), comorbidities of chronic obstructive pulmonary disease, chronic kidney disease, diabetes and malignancy, healthcare-associated infection risk factors hemodialysis, immune suppression and prior hospitalization, prior bacteremia, hospital length of stay prior to the onset of bacteremia, surgery, central line, total parenteral nutrition, septic shock, and infection source. APACHE, Acute Physiology and Chronic Health Evaluation. Area under the receiver operating characteristics curve = 0.738, Hosmer–Lemeshow *P* = 0.664.

**Table 5:** **Baseline and infection characteristics**

	**IAAT**		**Non-IAAT**		***P*** **value**
	***N***	**%**	***N***	**%**	
	819	76.97	245	23.03	
**Baseline characteristics**					
Age (years)					
Mean ± standard deviation	60.3 ± 15.1		61.8 ± 15.1		
Median (25th, 75th percentile)	62 (51, 71)		63 (52, 72)		0.165
Race					
Caucasian	543	66.30	159	64.90	0.643
African-American	218	26.62	71	28.98	
Hispanic	1	0.12	0	0.00	
Other	9	1.10	0	0.00	
Unknown	39	4.76	12	4.90	
Asian	9	1.10	3	1.22	
Sex, female	377	46.14	119	48.57	0.504
Admission source					
Home	585	71.52	123	50.20	<0.001
Nursing home (including LTAC)	62	7.58	30	12.24	
Transfer from other hospital	148	18.09	83	33.88	
Unknown	20	2.44	8	3.27	
Other	3	36.00	1	0.41	
Comorbidities					
CHF	150	18.32	64	26.12	0.009
COPD	190	23.30	73	29.80	0.043
CLD	134	16.36	36	14.69	0.619
DM	202	24.66	72	29.39	0.157
CKD	141	17.22	53	21.63	0.131
Malignancy	379	46.28	89	36.33	0.007
HIV	7	85.00	5	2.04	0.161
Charlson comorbidity score					
Mean ± standard deviation	5.00 ± 3.35		5.18 ± 3.52		
Median (25th, 75th percentile)	5 (2, 7)		4 (3, 8)		0.624
HCA risk factor					
Hemodialysis	55	6.80	38	15.64	<0.001
Immune suppression	334	41.70	90	37.34	0.228
Prior hospitalization	485	62.26	164	71.30	0.012
Nursing home residence	62	7.57	30	12.24	0.022
Prior antibiotics	429	52.38	170	69.39	<0.001
Hospital-acquired BSI^a^	366	44.69	137	55.92	0.002
Prior bacteremia within 30 days	95	11.60	39	15.92	0.074
**Sepsis characteristics**					
LOS prior to bacteremia (days)					
Mean ± standard deviation	7.0 ± 12.1		11.7 ± 19.6		
Median (25th, 75th percentile)	1 (0, 10)		5 (0, 16)		<0.001
Surgery					
None	575	70.90	162	66.94	0.033
Abdominal	152	18.74	36	14.88	
Extra-abdominal	84	10.36	44	18.18	
Central line	491	62.31	170	72.34	0.005
TPN at time of bacteremia or prior to it during index hospitalization	53	6.59	22	9.17	0.175
APACHE II score					
Mean ± standard deviation	16.5 ± 6.2		18.7 ± 6.6		
Median (25th, 75th percentile)	16 (12, 20)		18 (14, 22)		<0.001
Severe sepsis	451	55.07	108	44.08	0.003
Septic shock requiring pressors	368	44.93	137	55.92	
On mechanical ventilation	176	21.57	89	36.33	<0.001
Peak WBC					
Mean ± standard deviation	22.1 ± 18.3		22.9 ± 17.1		
Median (25th, 75th percentile)	17.0 (7.5, 33.8)		18.3 (8.6, 37.0)		0.298
Infection source^b^					
Urine	206	25.15	55	22.45	0.446
Abdomen	124	15.14	31	12.65	0.355
Lung	154	18.80	63	25.71	0.024
Line	87	10.62	22	8.98	0.548
CNS	6	0.73	1	0.41	1.000
Skin	41	5.01	21	8.57	0.043
Unknown	260	31.75	71	29.98	0.432
Polymicrobal BSI	130	15.87	59	24.08	0.003
MDR BSI	16	1.95	45	18.37	<0.001

^a^Hospital-acquired BSI defined as BSI that developed after day 2 of hospitalization. ^b^Multiple sources possible. APACHE, Acute Physiology and Chronic Health Evaluation; BSI, bloodstream infection; CHF, congestive heart failure; CKD, chronic kidney disease; CLD, chronic liver disease; CNS, central nervous system; COPD, chronic obstructive pulmonary disease; DM, diabetes mellitus; HCA, healthcare-associated; IAAT, initially appropriate antibiotic therapy; LOS, length of stay; LTAC, long-term acute care; MDR, multidrug resistant; TPN, total parenteral nutrition; WBC, white blood cells.

**Table 6:** **Distribution of inappropriate treatment by organism**

	**IAAT**		**Non-IAAT**	
	***N***	**%**	***N***	**%**
	819	76.97	245	23.03
*Pseudomonas aeruginosa*	129	75.44	42	24.56
*Acinetobacter* spp.	19	26.03	54	73.97
*Bacteroides* spp.	51	63.75	29	36.25
*Stenotrophomonas maltophilia*	2	9.09	20	90.91
*Enterobacteriaceae*				
*Klebsiella pneumoniae*	186	86.92	28	13.08
*Escherichia coli*	247	88.21	33	11.79
*Klebsiella oxytoca*	27	77.14	8	22.86
*Proteus mirabilis*	45	81.82	10	18.18
*Serratia marcescens*	39	84.78	7	15.22
*Citrobacter freundii*	22	88.00	3	12.00
*Enterobacter aerogenes*	29	82.86	6	17.14
*Enterobacter cloacae*	72	80.90	17	19.10
Polymicrobial	130	68.78	59	31.22

IAAT, initially appropriate antibiotic therapy.

**Table 7:** **Predictors of receiving initially inappropriate antibiotic therapy** ^**a**^

	**Odds ratio**	**95% confidence interval**	***P*** **value**
*Stenotrophomonas maltophilia*	91.981	20.538 to 411.956	<0.001
Multidrug resistant	23.045	12.097 to 43.900	<0.001
*Acinetobacter* spp.	17.410	9.600 to 31.574	<0.001
HIV	4.547	1.255 to 16.477	0.021
*Bacteroides* spp.	4.202	2.466 to 7.159	<0.001
Transfer from another hospital	2.280	1.527 to 3.403	<0.001
Polymicrobial	2.294	1.498 to 3.512	<0.001
Prior antibiotics	1.793	1.238 to 2.597	0.002
Congestive heart failure	1.683	1.097 to 2.582	0.017
APACHE II score	1.051	1.023 to 1.081	<0.001

^a^This model includes all factors identified in the model in Table 4 with the addition of the three pathogens with strikingly different initially appropriate antibiotic therapy patterns identified in Table 6. Please note that all other previously identified factors stayed in, except nursing home residence, which fell out based on significance (lower bound of the 95% confidence interval was 0.921). The area under the receiver operating characteristics curve is improved compared with the model in Table 4. However, we feel that this model does not add any clinical or policy utility when compared with the original model. While the multidrug-resistant designation provides an actionable data point with regard to stewardship and prevention of resistance development, the other microbiology variables simply represent organisms routinely isolated from septic patients. For this reason, we are offering this alternate regression and retaining the original regression as a part of the main manuscript. These data further emphasize the need for clinicians to know their individual centers’ case mix *vis-à-vis* microorganisms associated with sepsis and their predominant susceptibility patterns. APACHE, Acute Physiology and Chronic Health Evaluation. Area under the receiver operating characteristics curve = 0.827, Hosmer–Lemeshow *P* = 0.162.

## Discussion

This large retrospective analysis confirms that non-IAAT is a key determinant of short-term mortality among patients with severe sepsis and septic shock due to a Gram-negative organism. More importantly, our findings indicate that the presence of a MDR Gram-negative pathogen is strongly associated with non-IAAT. Despite the relatively low prevalence of a MDR phenotype among all subjects with Gram-negative bacteremia, these pathogens exert an excessive impact on mortality. In other words, MDR pathogens disproportionately affect outcomes through an intermediate step as it relates to antibiotic therapy. In light of the increasing frequency of multidrug resistance, our observations suggest that urgent action is needed to prevent potential escalation of mortality rates in severe sepsis and septic shock.

Because the co-occurrence of MDR pathogens and non-IAAT was relatively rare, it is important to consider the context of total non-IAAT exposure. The pool for the MDR pathogens as defined in our study comprises the vast majority of Gram-negative organisms responsible for serious infections in the ICU. That is, compared with *Acinetobacter* spp., for example, the relative prevalence of *P. aeruginosa* and *Enterobacteriaceae* was an order of magnitude higher. Epidemiologically, this imbalance makes it imperative for clinicians to consider these organisms first and foremost when choosing empiric treatment. We have demonstrated that multidrug resistance among these organisms comprises one important mechanism for errors in empiric coverage. At the same time, *Acinetobacter* spp. and *Stenotrophomonas maltophilia* infections, although a minority, were extremely likely to be subject to inappropriate empiric treatment (Table 6). Because the risk for drug resistance is very high among these organisms, the observed elevated rates of non-IAAT are probably not because the clinician did not consider their risk for resistance, but rather due to his/her determination that these were not likely pathogens. This approach therefore represents a slightly different mechanism for causing non-IAAT and implies a different solution. Rather than understanding the antibiogram of common pathogens, this requires a clinician to be aware of the rates of specific less common organisms at his/her institution. An additional important mechanism for receiving non-IAAT exists based on the timing of empiric therapy. Fully one-quarter of all non-IAAT fell into this category when there was no evidence of empiric treatment within 24 hours of obtaining the blood culture. This informs yet another corrective approach, one that requires simply to recognize the presence of a severe infection and to institute empiric treatment in a timely manner. These three mechanisms for exposure to non-IAAT and their corrective strategies are subtly yet importantly different from one another. In the current study we focus specifically on the impact of multidrug resistance on the risk of non-IAAT.

The prevalence of Gram-negative resistance has been mounting over the last decade [4-6]. However, most prior work describing the epidemiology of MDR Gram-negative pathogens has focused on the prevalence of resistance among specific species in specific infections. For example, a recent study demonstrated that between 2000 and 2009 nationwide in the United States there was a rise of MDR-PA from 10.7 to 13.5% in bloodstream infections, and from 19.2 to 21.7% in pneumonia [4]. The proportion of *P. aeruginosa* that met the MDR definition in the current study (15.0%) is consistent with these national estimates. The prevalence of carbapenem-resistant *Enterobacteriaceae* that we report here is also in line with national estimates [4-6]. In general, the similarity of the overall prevalence of multidrug resistance in our study to what has been reported nationally lends external validity to our observations. Moreover, our study is unique in its pragmatic perspective relevant to an ICU clinician and focuses on a common syndrome that represents a final common pathway for several infection types.

Much research from the last decade has highlighted the strong relationship between the choice of empiric antimicrobial treatment and the risk of death among patients hospitalized with serious infections. Most studies suggest that the risk of hospital death in association with non-IAAT goes up twofold to fourfold when compared with patients who receive appropriate coverage [7-9,11-15]. Furthermore, switching from inappropriate to appropriate coverage once the culture results have become available does not reduce the mortality risk imparted by this early failure [10]. In this way our study adds to the understanding of the importance of choosing appropriate empiric treatment specifically to the outcomes of Gram-negative sepsis, and extends this understanding to suggest not only the mechanism for this finding, but also the contribution of multidrug resistance to the risk of making this important error in early management.

The potential policy and public health implications of our results are significant. Most attempts to improve rates of IAAT have relied on a strategy of prompt administration of broad empiric coverage informed by the local antibiogram, followed by de-escalation. In fact, this is the strategy advocated by the Surviving Sepsis Campaign [16]. To prevent antibiotic abuse, the broad regimen is tailored as culture data become available, and the shortest appropriate course of therapy is given. This paradigm suggests that the way to address low rates of IAAT is to shift to using broader spectrum agents such as anti-pseudomonal carbapenems or ESBLs and/or chephalosporins. Unfortunately, in the case of MDR Gram-negative organisms, this is simply not an option. Few agents currently available provide *in vitro* activity against MDR-PA and CPE. Those agents that are available, such as colistin, carry important, albeit somewhat controversial, safety concerns [22-25]. Simply selecting broader spectrum agents for the initial therapy is therefore not an option, because the current antibiotic armamentarium does not cover these MDR organisms. This highlights why new agents are urgently needed. As such, regulatory authorities and policy-makers need to develop expedited pathways for antibiotic development and approval. Such initiatives in the United States as the Generating Antibiotic Incentives Now Act, which provide incentives to support the development of newer antibiotics, are to be lauded [26]. These efforts must continue to be expanded and refined.

An additional point worth emphasizing is the relatively low prevalence of MDR pathogens in our study, and the implications of this for potential overuse of empiric broad-spectrum antibiotics, if such are available. Although certainly suboptimal with respect to both overuse and increased resource utilization, at the moment there is no way to tailor such therapies with any degree of precision. Yet not administering appropriate coverage results in a high penalty for the patient who is unlucky enough to harbor a MDR organism, with a fourfold increase in the risk of death. This situation underscores the urgency of the need for development of faster diagnostic tools, as well as risk stratification algorithms that may help clinicians to use broad-spectrum drugs appropriately. At the moment, however, the only viable solution appears to be to understand local resistance patterns in real time and make therapeutic choices based on them.

Our study has a number of limitations. As a retrospective cohort it is prone to several forms of bias, most notably selection bias. We attempted to mitigate this by enrolling consecutive patients fitting the pre-determined enrollment criteria. Although we dealt with confounders by adjusting for those that were available, it is possible that some residual confounding remains. One specific potential residual confounder is the type of surgery; that is, although we have data on whether each patient either had a surgical procedure or was cared for on a surgical service during his/her hospitalization, we do not know whether the surgery was related to the sepsis episode or was performed for infectious source control. However, based on prior experience at BJC, only a minority of patients is likely to have undergone source control surgery. The fact that this is a single-center study in a very specific population of patients (those with Gram-negative sepsis) may diminish the generalizability of our results to other centers and populations. One important point is that Clinical Laboratory and Standards Institute break-points for susceptibility changed for some of the antibiotics during the study time frame [19,20]. The lowering of these values almost certainly resulted in an increase in the proportion of resistant organisms. This likely increase, however, would dilute rather than inflate the impact of multidrug resistance on the receipt of IAAT. Since we used only the susceptibility profile and the timing of antibiotic administration as surrogates for IAAT, our definition may have been overly liberal and included some cases that would have been deemed non-IAAT if other factors such as dosing and tissue penetration had been examined. Another source of possible misclassification is our use of ICD-9-CM codes to identify organ failures. While this identification may be less accurate than clinical data, this methodology has been validated and widely utilized in health services research [17]. The same situation arose for comorbidities, thus eliminating the possibility of examining whether or how their severity may impact the outcomes. Finally, because we examined hospital mortality rather than the more standard 28-day mortality as the primary outcome for our study, we may have underestimated the magnitude of this outcome.

## Conclusions

In summary, our study provides evidence that once the high risk of a serious infection has been recognized by a clinician and empiric treatment for common pathogens instituted, MDR organisms are an important factor in determining the risk of non-IAAT, and, by extension, hospital mortality in Gram-negative sepsis. Given the paucity of currently available antimicrobial options to cover this emerging threat, the key immediate solution is their prevention through various protocols to address ventilator and central venous catheter care, as well as through antibiotic stewardship programs [27-29].

### Definitions

- Septic shock: vasopressors (norepinephrine, dopamine, epinephrine, phenylephrine, or vasopressin) initiated within 24 hours of the blood culture collection date and time.
- IAAT: initially prescribed antibiotic regimen active against the identified pathogen based on *in vitro* susceptibility testing and administered within 24 hours following blood culture collection.
- Prior antibiotic exposure: any exposure to an antibiotic within the preceding 90 days.
- Prior hospitalization: any hospitalization within the preceding 90 days.
- Prior bacteremia: a bacteremia episode within 30 days of the current episode.
- MDR-PA: a *P. aeruginosa* resistant to at least three of the following classes of antimicrobials: aminoglycosides, anti-pseudomonal penicillins, anti-pseudomonal cephalosporins, carbapenems, and fluoroquinolones.
- MDR case: blood culture positive for a MDR-PA, an ESBL organism or a CPE.
- Healthcare-associated infection: the presence of at least one of the following risk factors: recent hospitalization (within 90 days of the current one); immune suppression; nursing home residence; hemodialysis; and prior antibiotics (within 90 days of the current hospitalization).

## Key messages

- Among patients with severe sepsis/septic shock due to a Gram-negative organism, initially inappropriate antibiotic treatment is associated with a threefold increase in hospital mortality.
- Multidrug resistance is strongly associated with inappropriate treatment.

## Abbreviations

CPE: carbapenemase-producing *Enterobacteriaceae*
ESBL: extended-spectrum beta-lactamase
IAAT: initial appropriate antibiotic therapy
ICD-9-CM: International Classification of Diseases, Version 9, Clinical Modification
MDR: multidrug resistant
MDR-PA: multidrug-resistant *Pseudomonas aeruginosa*
